# Impaired Set-Shifting from Dorsal Stream Disconnection: Insights from a European Series of Right Parietal Lower-Grade Glioma Resection

**DOI:** 10.3390/cancers13133337

**Published:** 2021-07-03

**Authors:** Suzanne L. Hartung, Emmanuel Mandonnet, Philip de Witt Hamer, Martin Klein, Michel Wager, Fabien Rech, Johan Pallud, Catarina Pessanha Viegas, Sebastian Ille, Sandro M. Krieg, Pierre A. Robe, Martine J. E. van Zandvoort

**Affiliations:** 1 Department of Neurology and Neurosurgery, University Medical Center Utrecht, 3584 CX Utrecht, The Netherlands; p.robe@umcutrecht.nl (P.A.R.); M.j.e.vanzandvoort@umcutrecht.nl (M.J.E.v.Z.); 2 Department of Neurosurgery, Lariboisiere Hospital, 75010 Paris, France; emmanuel.mandonnet@aphp.fr; 3 Department of Neurosurgery, Location VUmc, Cancer Center Amsterdam, Amsterdam UMC, 1081 HV Amsterdam, The Netherlands; p.dewitthamer@amsterdamumc.nl; 4 Department of Medical Psychology and Brain Tumor Center Amsterdam at Amsterdam UMC, Vrije Universiteit Amsterdam, 1081 HV Amsterdam, The Netherlands; m.klein@amsterdamumc.nl; 5 Department of Neurological Surgery, Poitiers University Hospital, 86021 Poitiers, France; michel.wager@chu-poitiers.fr; 6 CHRU-Nancy, Service de Neurochirurgie, Université de Lorraine, F-54000 Nancy, France; fabien.rech@orange.fr; 7 CNRS, CRAN, Université de Lorraine, F-54000 Nancy, France; 8 Department of Neursurgery, Saint-Anne Hospital, 75014 Paris, France; j.pallud@ghu-paris.fr; 9 Department of Neurosurgery, Hospital Garcia de Orta, 2805-267 Almada, Portugal; catarina.viegas@jmellosaude.pt; 10 Department of Neurosurgery, School of Medicine, Klinikum rechts der Isar, Technical University Munich, 80333 Munich, Germany; Sebastian.Ille@tum.de (S.I.); sandro.krieg@tum.de (S.M.K.); 11 Department of Experimental Psychology, Utrecht University, 3584 CS Utrecht, The Netherlands

**Keywords:** cognitive flexibility executive function, glioma, Trail Making Test, connectivity, brain network, intraoperative monitoring

## Abstract

**Simple Summary:**

Awake surgery with cognitive monitoring has increasingly been implemented to preserve brain networks and functionality. More recently, not only surgery in the left but also in the right hemisphere, i.c., the parietal lobe, was associated with potential risk for deficits in cognitive functions, such as cognitive flexibility. We describe an explorative pilot study in an international consortium within clinical care as usual. Careful interpretation of our findings indicates that disconnection of the lateral part of the dorsal stream correlated with impaired set-shifting. More importantly, it illustrates the need for international collaboration on neuropsychological tests and methodologies to improve our understanding of white matter networks at risk during awake surgery.

**Abstract:**

Awake surgery with cognitive monitoring has increasingly been implemented to preserve brain networks and functionality. More recently, not only surgery in the left but also in the right hemisphere, i.c., the parietal lobe, was associated with potential risk for deficits in cognitive functions, such as cognitive flexibility. In this explorative pilot study, we compare cognitive performance more than three months after surgery with baseline measurements and explore the association between cognitive decline and subcortical tracts that may have been severed during surgery in the right hemisphere. Twenty-two patients who underwent surgery for a right parietal low-grade glioma were assessed pre- and postoperatively using the Trail Making Test and the Stroop task to administer set-shifting abilities and inhibition. Volume measurements and lesion–symptom mapping analyses were performed on postoperative MRI scans. Careful interpretation of the results shows a change in TMT performance and not on the Stroop Task when the lateral part of the arcuate fasciculus is damaged, indicating that disconnection of the lateral part of the dorsal stream might be correlated specifically with impaired set-shifting and not with inhibition. More importantly, this study underlines the need for international concertation to allow larger studies to increase power and perform more detailed analyses.

## 1. Introduction

Monitoring cognition has become imperative in glioma surgery to preserve cognitive functions and maintain quality of life. Although glioma surgery in both the left and right hemispheres is part of clinical practice, monitoring cognition has been more often applied to eloquent areas in the left hemisphere, with the main focus on motor abilities and language skills [1]. From a network perspective, the whole brain is eloquent due to the connections of white matter tracts, which makes cognitive monitoring in the right hemisphere equally desirable [2]. However, our knowledge of the relationship between cognition and glioma in the right hemisphere is largely based on case reports, and uniformity in cognitive monitoring lags behind. While we have learned about cognitive functions such as visuospatial abilities, visuoperceptual abilities and language capabilities of the right hemisphere, a prime example in the face of cognitive functions that is overlooked, is cognitive flexibility [3]. Yet this function should be monitored. The function receives increasingly more attention during awake glioma surgery since the risk of postoperative deficits in cognitive flexibility can affect the patient’s quality of life considerably, but the architecture of the function in the brain remains ambiguous [4]. Barbey, Colom and Grafman (2013) suggest that a set of fronto–parieto–temporal connections primarily within the left hemisphere are to be associated with cognitive flexibility [5]. However, recent reports have challenged this unilateral predominance. A case report illustrates that focal lesions in white matter can generate a decline in cognitive flexibility after surgical resection of a low-grade glioma in the right supramarginal gyrus [6]. Consonantly, alterations were found in the connectivity of the fronto–parieto–temporal network, with damage to the superior longitudinal fasciculus III, the long segment of the arcuate fasciculus and the posterior part of the arcuate fasciculus; this posterior part is referred to as the vertical temporoparietal fasciculus in new nomenclature [7]. Dajani and Uddin (2015) support this finding by suggesting that the right hemisphere may be recruited with cognitive development since increased activation in right-lateralized regions and decreased activation in left-lateralized regions are seen with age in cognitive flexibility tasks [8]. This is supported by studies that show the central role of the right parietal lobe in the integration of information, as it is considered to be a network hub [9,10].

Unravelling the brain–cognition relationship of cognitive flexibility—and specifically confirming the potential implication of the superior longitudinal fascicle III (SLF III), the longitudinal part of the arcuate fascicle (AF longitudinal) and the posterior part of the arcuate fascicle (AF posterior)—can further benefit from lesion-based brain–behaviour studies. In this paper, we analysed a series of clinically enrolled patients operated for a right parietal lower-grade glioma collected through a multicentred international collaboration. We compare cognitive performance more than three months after surgery with baseline measurements and explore the association between cognitive decline and subcortical tracts that may have been severed during surgery.

## 2. Methods

### 2.1. Ethics Statement

The Medical Research Ethics Committee confirms that the Medical Research Involving Human Subjects Act (WMO) does not apply to this retrospective study (protocol number 20-727/C) and that therefore an official approval of this study by the MREC Utrecht is not required under the WMO.

### 2.2. Participant Data

The eligibility criteria included patients who were at least 18 years old, who had a primary lower-grade glioma (WHO II and III) located in the right parietal lobe and who underwent first-ever surgical resection. All subjects were operated between 2010 and 2017 in one of the eight contributing hospitals across Europe. Availability of anatomical preoperative and postoperative MRI scans and neuropsychological data was mandatory for inclusion in the study.

### 2.3. Cognitive Data 

#### 2.3.1. Neuropsychological Assessment

Based on a consensus meeting between the collaborative centres, the common tasks associated with cognitive flexibility which were administered in all centres as part of the clinical-care-as-usual were the Trail Making Test (TMT) and the Stroop Color-Word Test (SCWT) [11,12]. The TMT consists of two parts: part A requires the patient to draw a line to connect 25 spatially distributed encircled numbers in numerical order as quickly as possible. In part B, the patient connects numbers and letters in numerical and alphabetical order while alternating between the numbers and letters (e.g., 1-a-2-b-3-c, etc.) [13]. The total time of completion of each part is the direct score of the task. Part A reflects motor and perceptual speed, whereas part B is linked to several parts of executive function, such as set-shifting, task-shifting, inhibition and working memory [14,15]. The purest index of cognitive flexibility—and, more precisely, set-shifting—is represented by the derived score of the B-to-A ratio since this score eliminates the attentional speed effect measured in part A [16,17]. In addition, the SCWT also measures cognitive flexibility but is more strongly associated with inhibition and working memory [18]. The test has been included to provide convergent evidence for the specificity of the relatively pure measure of cognitive flexibility by the TMT and associated white matter tracts. Multiple versions have been developed, but the basic paradigm remains the same. The most commonly used version in this study consists of three subtasks [12]. The first subtask consists of 100 colour words (yellow, green, blue and red) printed in black ink, which are arranged in random order. The second subtask displays 100 rectangular coloured patches, again arranged in random order. The task here is to correctly read either the word on subtask 1 or name the colour patch on subtask 2. The third subtask consists of colour words printed in an incongruent ink colour (e.g., the word “blue” is printed in yellow ink) [19]. Unlike the first two subtasks, the third subtask requires an individual to suppress the automatic reading of the word and instead name the incongruent colour of the ink [20]. The score that best represents cognitive flexibility in the SCWT is the derived interference score, where the attentional speed effect is cancelled out by subtracting the average time of cards 1 and 2 from the time on card 3.

#### 2.3.2. Analyses of the Neuropsychological Data

z-scores were calculated to analyse neuropsychological data. Normative scores from published norming studies were used for each cognitive test to compare results with outcomes from healthy adults. As for the TMT norm scores, Arbuthnott and Frank (2000) calculated ratio scores, with a mean of 2.6 (*SD* = 1.1), from a sample of 34 healthy adults aged from 18 to 48 years [17]. Raw TMT ratio scores were used to calculate derived z-scores as [(preoperative TMT ratio − mean)/*SD*]. Results on the SCWT were compared to norms scores from a total sample of 987 people aged from 49 to 81 years [21]. The SCWT-derived contrast z-scores were calculated as [completion time in seconds on card 3—[{completion time in seconds on card 1 + completion time in seconds on card 2}/2], with a mean of 50.4 (*SD* = 19.7) [22]. To align results, derived z-scores were recoded. Thus, a decline in performance was presented as a negative z-score. Change in performance in an individual after surgical resection of the low-grade glioma was measured by calculating the change index, that is, [postoperative TMT recoded z-score -preoperative TMT recoded z-score] and [postoperative SCWT recoded z-score - preoperative SCWT recoded z-score]. To define a clinically significant change between preoperative and postoperative performance, we employed a liberal 68% confidence interval [23]. In other words, change index scores greater than one standard deviation or smaller than minus one standard deviation indicate a change in the individual’s preoperative and postoperative performance.

### 2.4. Imaging Data

Magnetic Resonance Image (MRI) data were acquired sagittally pre- and post-surgery. Based on the availability and quality of the images, the protocol included T1-weighted 3D images, T2-weighted 3D images and FLAIR-weighted 3D images.

### 2.5. Image Analyses

Image files in DICOM format were converted to NIFTI files using the dcm2nii application in MRIcron. Pre-processing steps were performed on each postoperative MRI scan before conforming the data to the standardized Montreal Neurological Institute (MNI) space, using the Clinical Toolbox in SPM12 [24], implemented in MATLAB [25]. Spatial segmentation was performed by a skilled operator and checked by a neurosurgeon using ITK-SNAP software [26]. This involved masking the area of resection and residual glioma tissue by masking segmentation to prevent the lesion area from contributing to the normalization process [27]. In addition, resection segmentation was performed to outline the resection area.

Imaging data were analysed by using two complementary techniques. Firstly, resection volumes were measured in ITK-SNAP to determine the extent of resection. Secondly, lesion–symptom mapping was performed, involving overlay plots using the MRIcron program [28]. To map the areas of interest with respect to the white matter tracts of interest, an atlas was downloaded from http://toolkit.bcblab.com (accessed on 1 June 2019), which provided tractography of the SLF III and the longitudinal and posterior parts of AF based on high-resolution diffusion-weighted imaging datasets [29]. These white matter tract segments were, for each patient, co-registered with the resection cavities on the atlas. For each white matter tract segment, two categories were defined (lesioned at surgery or not) and patients were allocated to these categories. Within each group, the distributions of the delta Z score (change index) of these patients prior to and after surgery for each test were compared using non-parametric Mann–Whitney tests for independent samples.

## 3. Results

### 3.1. Demographic Data

In this explorative pilot study, thirty-six patients in which the parietal lobe was the main locus of the low-grade primary glioma were sent in for inclusion from the collaborating centres. Twenty-two patients fulfilled the entry criteria (11 female and 11 male; median 39 years of age). Patients with a first-ever LGG were included when both preoperative and postoperative neuropsychological assessments with the TMT and/or the Stroop were present and the imaging data were of sufficient quality. As part of routine clinical practice, all patients were tested for the presence of neglect at the time of neuropsychological assessments. Based on clinical interpretation, none of the included patients were preoperatively or postoperatively diagnosed with neglect. The collaboration was between eight hospitals across Europe: Centre Hospitalier Saint-Anne (Paris, France) (*n* = 3), Hospital Lariboisière (Paris, France) (*n* = 3), Centre Hospitalier Universitaire de Poitiers (Poitiers, France) (*n* = 2), Centre Hospitalier Régional Universitaire de Nancy (Nancy, France) (*n* = 1), Klinikum rechts der Isar der Technischen Universität München (München, Germany) (*n* = 1), Hospital Garcia de Orta (Lisbon, Portugal) (*n* = 2), Amsterdam University Medical Centre (Amsterdam, the Netherlands) (*n* = 5) and University Medical Centre Utrecht (Utrecht, the Netherlands) (*n* = 5). Patients’ clinical characteristics are summarized in Table 1.

### 3.2. Neuropsychological Outcome

The preoperative neuropsychological evaluation was within (1–10 days) and postoperatively between (3–18 months). In Table 2, pre- and postoperative test results of the TMT ratio scores and the SCWT contrast scores can be found calculated into z-scores. Five patients performed worse on the postoperative assessment on the TMT in comparison to the preoperative assessment, expressed in the TMT change index (cases A, H, L, U and V). No improvement in TMT performance after glioma surgery was discerned. On the SCWT, two patients declined in performance and one patient (I) improved after glioma surgery.

### 3.3. Lesion Symptom Mapping Analysis

**Figure 1:**
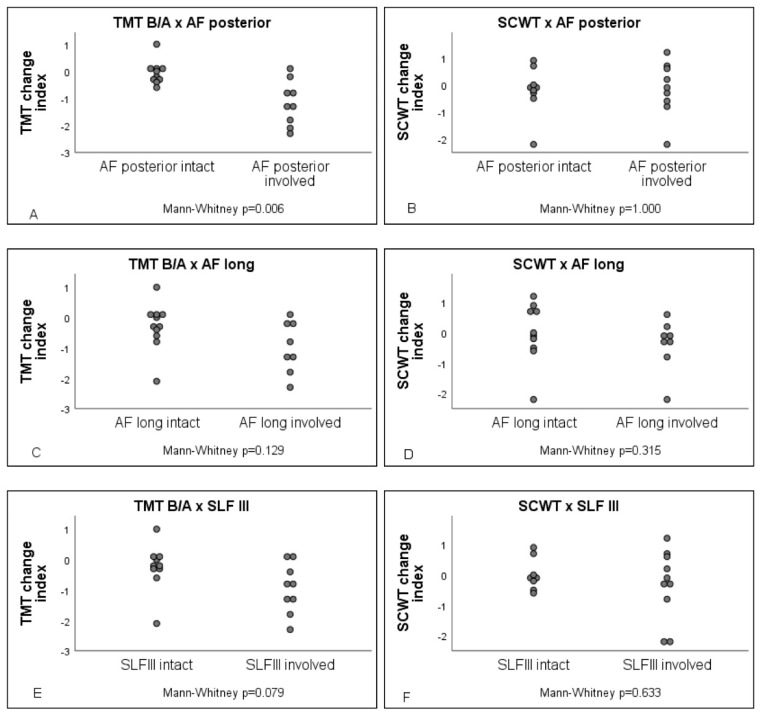
TMT B/A and SCWT change index in relation to white matter tracts. Involvement of the posterior part of AF in (**A**) TMT performance and in (**B**) SCWT performance; involvement of the longitudinal part of AF in (**C**) TMT performance and (**D**) SCWT performance; involvement of the SLF III in (**E**) TMT performance and (**F**) SCWT involvement.

**Figure 2:**
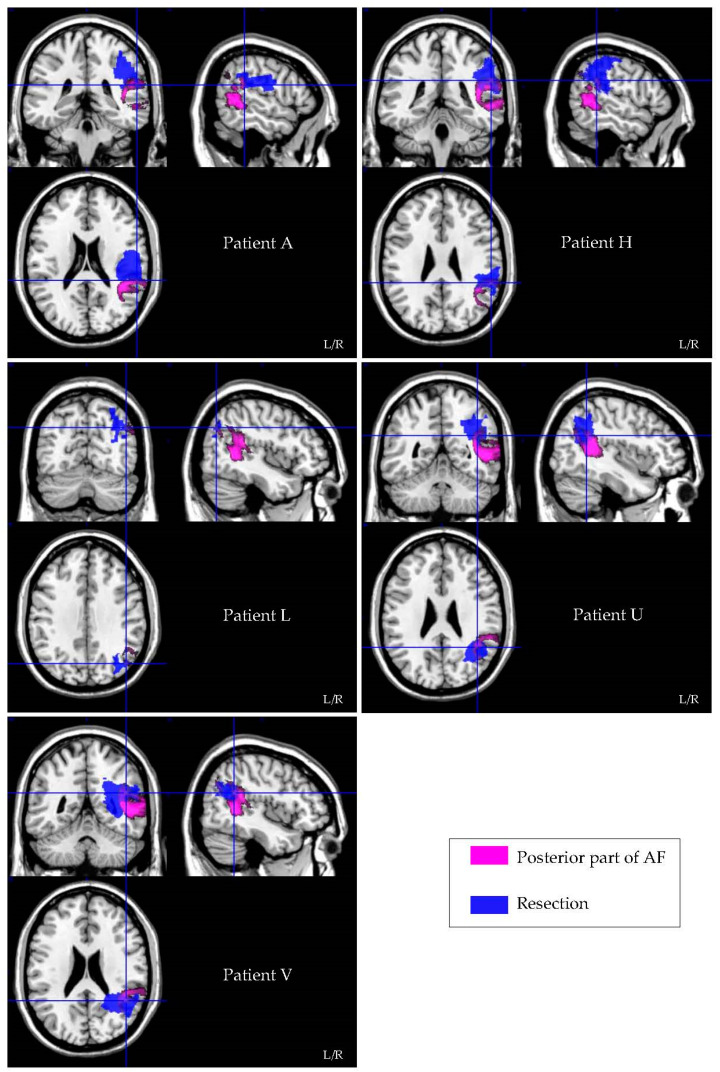
Colour-encoded white matter tract (posterior part of AF = pink) incorporated in individual resection overlays (resection = blue) of patients who declined in TMT B/A performance.

Patients with versus without damage to the posterior part of the AF performed significantly worse on the TMT B/A ratio (U = 12, AUC = 0.133, *p* = 0.006), see Figure 1. The level of significance did not differ after the exclusion of the patients who were seen 12–18 months postoperatively, suggesting that the finding was not biased by the time between surgery and postoperative neuropsychological assessment. In addition, Fisher’s exact test revealed that cognitive decline does not differ between radiotherapy (*p* = 0.351) or rehabilitation (*p* = 0.646). Figure 2 visualizes the resection–symptom overlay of five patients who performed worse on the TMT B/A ratio after surgery. No difference in performance on the TMT B/A ratio was found for damage to the longitudinal part of AF (U = 25, *p* = 0.129) or the SLF III (U = 23, *p* = 0.079). Looking at the same predefined white matter tracks, performance on the SCWT appeared not to differ between those affected versus those patients in whom the tracks were not affected (AF posterior U = 40.5, *p* = 1.000; AF longitudinal U = 28.5, *p* = 0.315; SLFIII U = 34.5, *p* = 0.633).

### 3.4. Resection Volume Measurements

Resection volumes varied widely, between 2.74 cm^3^ and 89.80 cm^3^ (*Mdn* = 17.42 cm^3^). Interestingly, the decrease in the TMT B/A ratio was found in the patients with smaller resection volumes, 6.36 cm^3^ and 26.87 cm^3^, and not in the larger resection volumes (47.99–89.80 cm^3^). These results suggest that resection volume is not directly linked to a decline in performance on the TMT B/A ratio after surgery.

## 4. Discussion

In this pilot study, we set out to explore the risk for a decrease in cognitive flexibility after surgery in patients with a low-grade glioma in the right parietal lobe through an international collaboration within clinical care as usual. We did find careful support that white matter tracks in the right parietal lobe subserve cognitive flexibility and, as such, should be handled with the utmost care during tumour surgery. Moreover, this relation appeared specific to the set-shifting abilities and the posterior part of the AF. However, this relation is based upon small numbers, and only a minority of the patients were left with a decrease in task performance associated with cognitive flexibility. This study underlines the need for international concertation to allow larger studies to increase power and perform more detailed analyses.

In the patients admitted for this retrospective study, the TMT and the Stroop test were found to be the tasks administered to test cognitive flexibility. By interpreting the change index instead of individual z-scores, we eliminated a possible bias created by the influence of age and education on cognitive performance. First, a non-parametric analysis revealed the association between a decline in performance on the TMT B/A and damage to the posterior part of AF after glioma surgery. This supports a link between damage to the posterior part of AF and set-shifting. Where both the TMT and SCWT assess the construct of cognitive flexibility, they are not merely different measures of the same construct since they also measure non-overlapping variance [30]. Ultimately, the specificity of the relationship between the TMT and the posterior part of AF was demonstrated by the dissociative relationship between performance on the SCWT, measuring more specifically inhibition, and damage to the posterior part of AF.

Second, and in addition to the growing evidence suggesting that a greater extent of resection of low-grade gliomas improves long-term survival and delays the reoccurrence of the glioma [31], our findings showed no decline in cognitive flexibility when a more extensive resection was performed. In fact, two patients with relatively large resection volumes showed above-average performance on neuropsychological tests pre- and post-surgery. This implicates that cognitive inflexibility is not merely related to the extent of resection, strengthening the necessity of plasticity-induced functional reshaping in the brain to comprehend neuropsychological outcomes after surgery.

The necessity to preserve cognitive flexibility for social interactions, personal development and dealing with life events is recognized in the field of neurosurgery. Cognitive flexibility falls under the scope of executive function, allows for adaptation in response to changes in the environment and is seen as a multifarious construct [5]. It enables an individual to identify what is in flux, to become aware of an old insufficient strategy, to inhibit the previous responses and to reconfigure a new strategy [5,8]. Having greater cognitive flexibility is beneficial across the lifespan. In early and middle childhood, cognitive flexibility contributes to academic achievements and predicts better development in math and reading skills [32,33]. While cognitive flexibility is still developing throughout adolescence and early adulthood, drastically changing life events demand high cognitive flexibility to adjust to the new requirements that arise in adolescence. Failure to flexibly adjust may result in social exclusion, limited professional development and psychiatric disorders [34]. In adulthood, cognitive flexibility is associated with the ability to recover from negative life events and being more resilient to minor stressors, increasing the quality of life [35].

From a clinical perspective, these results suggest a role for testing cognitive flexibility intraoperatively in the right hemisphere during glioma surgery. We indeed show that resection in the right parietal lobe might lead to deficits of cognitive flexibility, a crucial cognitive function for independent and social life [34]. In the absence of specific rehabilitation for this cognitive function and in agreement to a recently proposed guideline for the development of novel intraoperative neuromonitoring techniques [36], its specific intraoperative monitoring deserves to be developed [1]. In addition, such intraoperative testing, in the setting of awake craniotomies, will refine our insight into brain–behaviour relationships [1]. With respect to intraoperative monitoring, our results suggest that functions other than language and motor functions should be mapped and that the use of one test alone, such as the SCWT, will not be discriminatory enough to predict specific cognitive flexibility deficits during awake glioma surgery [37].

Parietal location is very rare in lower-grade glioma [38]. The strength of our explorative approach resides in an international inclusion of clinically enrolled patients. This collaborative effort allowed us to gather the largest series for lesion–symptom mapping in right parietal lower-grade glioma. Howbeit, the current study is a prelude for multicentre research in glioma populations since great heterogeneity is seen in followed procedures between centres that progressively perform awake craniotomies in the right parietal lobe. Pursuing procedural coherence will benefit further research in large populations and will ultimately improve clinical practice.

Again, the current results should be interpreted with caution given the explorative nature of this study, the relatively small sample size limiting the statistical power and the generalizability of our findings. In particular, we cannot exclude a role for the right SLFIII and/or long AF in cognitive flexibility. Aside from white matter, correlations between grey matter and set-shifting abilities should also be given attention [39]. Direct electrical stimulation during awake glioma surgery should be used as a converging method to obtain comprehensive insight into the role of both cortical and subcortical regions, critical for set-shifting abilities. Furthermore, in our cohort, radiotherapy and rehabilitation did not influence cognitive outcome; however, the impact of postoperative seizures, ischemia or chemotherapy could not be statistically analysed due to the small sample size. Yet these influences should be taken into account considering the link between neurocognitive deficits and factors such as additional neuro-oncological treatments, seizures and antiepileptic drugs [40]. It is this knowledge of inevitable potential hazards in these patients that makes it all the more important to try to avert cognitive loss from resection. By performing resection plots and volume measurements, our approach was built upon derived and simulated white matter tractography instead of actual pre- and postoperative DTI brain scans of the patients. Follow-up and confirmatory studies on the value of testing of cognitive flexibility by our consortium will be performed using this technique [41,42,43].

## 5. Conclusions

Taken together, the results of the explorative pilot study demonstrate that cognitive flexibility, measured by the TMT B/A ratio, can decline after surgical resection of a lower-grade glioma in the right parietal lobe. Specifically, the posterior part of AF could be carefully associated with a decline in set-shifting, as measured by the TMT B/A ratio performance. However, the possible contribution of postoperative treatments should be recognized and considered in further research. In addition, the volume of resection appeared not to be linked to a decline in cognitive flexibility, showing that the preservation of function does not necessarily coincide with less radical resections. These results pave the way to additional studies in international concertation on the benefits of intraoperative cognitive monitoring of this cognitive function in glioma surgery, especially in the right parietal lobe. Implementing well-attuned procedures across centres will forward quantitative comparison and will improve both clinical practice and research.

## Figures and Tables

**Table 1:** Patients’ characteristics.

Patient	Age	Sex	Months *	Radiotherapy *	Chemotherapy *	Rehabilitation *	Seizures *	Start AED *
A	47	M	6	N	N	N	N	N
B	36	F	4	Y	N	N	Y	Y
C	31	M	9	N	N	N	N	N
D	34	M	6	Y	N	N	N	N
E	55	M	6	N	N	Y	N	Y
F	33	F	3	Y	N	N	N	N
G	21	M	3	N	N	N	N	N
H	40	F	4	N	N	Y	N	N
I	53	M	4	N	N	Y	N	N
J	36	M	4	N	N	Y	N	N
K	31	F	3	N	N	Y	N	N
L	67	F	3	N	N	Y	N	N
M	49	F	3	N	N	Y	N	N
N	39	F	12	Y	N	N	Y	Y
O	34	M	15	Y	N	Y	N	N
P	23	F	11	Y	N	Y	N	N
Q	39	F	17	Y	N	N	N	N
R	48	F	18	Y	Y	Y	N	N
S	30	M	3	N	N	N	N	N
T	48	M	4	N	N	N	N	N
U	59	F	5	N	N	N	Y	Y
V	49	M	3	N	N	N	N	N

* Between date of surgery and postoperative neuropsychological assessment.

**Table 2:** Results from neuropsychological assessment.

	TMT			SCWT		
	Preoperative *	Postoperative *	Change Index	Preoperative *	Postoperative *	Change Index
A	+1.0	−0.3	−1.3	+1.3	+1.0	−0.3
B	+0.3	+0.4	+0.1	+1.0	+0.7	−0.3
C	+1.1	+0.9	−0.2	+1.8	+1.7	−0.1
D	+0.5	+0.2	−0.3	+0.6	+0.1	−0.5
E	−0.6	+0.4	+1.0	-	+0.2	-
F	+0.3	+0.3	0.0	+0.4	+0.3	−0.1
G	+0.1	−0.8	−0.8	−0.6	+0.1	+0.7
H	−0.1	−1.9	−1.8	−0.2	−2.4	−2.2
I	+0.3	+0.4	+0.1	−1.8	−0.7	+1.2
J	+1.0	+0.7	−0.3	−1.0	−0.1	+0.9
K	−0.1	−0.8	−0.8	−0.3	−1.2	−0.8
L	−1.2	−3.3	−2.1	+0.7	+0.1	−0.6
M	+0.2	+0.3	+0.1	+0.8	+0.6	−0.2
N	−0.1	−0.5	−0.4	+0.2	−1.9	−2.2
O	+0.9	+0.3	−0.6	+0.5	+1.2	+0.7
P	+0.4	+0.5	+0.1	+0.8	+0.8	+0.0
Q	-	-	-	+1.1	+1.0	−0.1
R	-	-	-	+2.2	+2.4	+0.2
S	-	-	-	+0.1	+0.7	+0.6
T	+0.5	+0.3	−0.2	-	-	-
U	−2.6	−3.9	−1.3	-	-	-
V	+1	−1.3	−2.3	-	-	-

Data are z-scores (* recoded).

## Data Availability

Data Transfer Agreements, between UMC Utrecht and Data Providers from eight contributing centres across Europe, state that all Data remains the sole property of the Data Providers. UMC Utrecht and Data Providers use appropriate safeguards to prevent use or disclosure of the Data other than as permitted under the Data Transfer Agreements.
